# The Importance of Positive Psychological Factors among People Living with HIV: A Comparative Study

**DOI:** 10.3390/bs12080288

**Published:** 2022-08-15

**Authors:** Cristina Rivera-Picón, María Hinojal Benavente-Cuesta, María Paz Quevedo-Aguado, Pedro Manuel Rodríguez-Muñoz

**Affiliations:** 1 Faculty of Health Sciences, Nursing, Pontifical University of Salamanca, 37002 Salamanca, Spain; 2 Faculty of Nursing and Physiotherapy, University of Salamanca, 37008 Salamanca, Spain; 3 Departament of Nursing, Instituto Maimónides de Investigación Biomédica de Córboda, 14005 Córdona, Spain

**Keywords:** nursing, HIV, psychological well-being, resilience, coping strategies, adaptation

## Abstract

We aim to identify the differences in psychological well-being, resilience, and coping strategies between healthy subjects and HIV patients. The design followed in this work was empirical, not experimental, and cross-sectional with a correlational objective. The sample included a total of 399 participants (199 patients with HIV and 200 without pathology). The instruments applied for data collection were as follows: a questionnaire on socio-demographic data, the Psychological Well-being Scale, the Resilience Scale and the Coping Strategies Questionnaire. The study period was from February 2018 to January 2020. Patients with HIV had a significantly lower score than healthy subjects, in the resilience factors of perseverance and self-confidence. Subjects with HIV scored less in all dimensions of psychological well-being, with the exception of the dimension of autonomy. Finally, it was observed that HIV-positive subjects used rational coping strategies less frequently than healthy subjects, based on social support seeking and problem-solving coping. However, HIV patients scored higher in emotional coping strategies than healthy individuals.

## 1. Introduction

Human immunodeficiency virus (HIV) infection and AIDS diagnosis are currently a global public health problem. This pathology, in addition to producing a significant burden of disease in terms of morbidity and mortality, has had an enormous impact on the demography and economy of the countries most affected [1]. Currently, estimates provided by WHO and UNAIDS in 2017 state that 36.7 million people worldwide were diagnosed with HIV at the end of 2016. In the same year, approximately 1.8 million subjects were infected [1]. In Spain, the latest data show that, until 30 June 2019, 3244 new cases of HIV were detected [2].

The history of HIV infection is broad, not only scientifically, but also sociologically, in the way that it has impacted society [3]. Thus, the diagnosis of this pathology has complex psychological and social repercussions, which apparently hinder the well-being of these patients; thus, it is necessary to develop a process of adaptation to the disease [4,5,6]. 

HIV infection is a highly complex pathology, with a multifactorial process, which must be addressed from a biopsychosocial model. The subjects living with this disease deal with numerous physiological, socio-cultural, economic and psychological stressors that constitute a potential threat to their physical and mental health [7,8]. 

In this way, the social stigma associated with the diagnosis of HIV is considered a significant threat to combating the disease [9]. In addition, previous studies show that stigma is associated with more severe disease symptoms such as fatigue, gastrointestinal discomfort, numbness or body changes. It is even stated that people living with pathologies with clear social stigma suffer more perceived psychological stress [10,11]. Such stress, in turn, is negatively related to health, increasing the most severe HIV symptoms, the progression of the disease and altering the CD4 count and an increased risk of mortality and development of AIDS [4].

Because the stigma of HIV is difficult to eliminate but continues to have negative implications for the health of these patients, it is essential to identify strategies to protect against the harmful effects of this stigma. Therefore, we consider it essential to carry out research in this regard, in order to determine the resilience, psychological well-being and coping strategies most used in these patients [10].

In this line, resilience is defended as a dampening variable of physical and mental health, relating to a better quality of life and adaptation to the disease [12]. Due to its importance in the health of the subjects, the influence of resilience has been studied in different chronic pathologies such as cancer, diabetes, rheumatic diseases, rheumatoid arthritis, chronic pain, multiple sclerosis, HIV, urinary incontinence, renal insufficiency and cardiovascular diseases [13,14,15]. The common denominator of these studies is to consider that individuals with higher levels of resilience have less depression, anxiety and somatisation, which are common conditions in patients with chronic diseases such as HIV [16,17,18]. In addition, resilient people are better able to cope with the processes of illness, both their own and others, coming out strengthened from the situation [19,20,21,22,23,24].

In relation to psychological well-being, we also find data on its relationship with biological indicators associated with health. It has been shown that optimal levels of psychological well-being improve the immune capacity of individuals and healthy behaviours. In addition, subjects with a better psychological state benefit from an easier and faster recovery from the disease [25]. In this way, we consider it important to know the pathologies associated with lower welfare levels.

Finally, based on the last variable studied in this project, it is highlighted that coping strategies are described as possible protective factors of health and as promoters of psychological well-being [26]. Thus, it is evident that coping is a mediating factor that allows us to adapt to the disease. Therefore, it is essential that patients develop effective coping strategies to meet the demands of different diseases.

Studies carried out with patients diagnosed with HIV show that avoidance-based coping does not protect against psychological distress, promoting levels of stress and anxiety [27]. Thus, the most active coping strategies, characterised by the mobilisation of the patient to deal with the disease, are associated with greater well-being. In addition, in a study by Milena et al. (2009) [28], which evaluated the relationship of coping strategies with anxiety and depression in 92 subjects diagnosed with HIV, showed that the use of problem-solving coping, positive reappraisal, social support seeking and religious coping were associated with lower levels of anxiety and depression. 

Additionally, some studies reflect that resilience and more active coping strategies help to face the stigmatization of the disease, changing the beliefs of patients towards it and improving the psychological well-being of patients [4,29]. 

Thus, the positive impact on the health of HIV patients with high levels of psychological well-being and resilience, as well as the use of active coping strategies, was studied. Based on the theoretical aspects stated, which reflect the difficulties associated with the diagnosis of this disease, we consider it important to determine whether there are differences in resilience, psychological well-being and coping strategies among HIV carriers and healthy participants. This can be of great importance to nursing staff in terms of creating secondary prevention plans, focusing on the psychological aspects most frequently affected in HIV patients.

## 2. Materials and Methods

### 2.1. Aims and Design of the Study

The aim of this study was to identify the differences in psychological well-being, resilience, and coping strategies between healthy subjects and HIV patients. Thus, our intention was to determine if subjects with HIV show a different psychological pattern in relation to resilience, psychological well-being and the use of coping strategies compared to HIV-negative subjects. The study had a non-experimental cross-sectional design with a correlational objective. 

### 2.2. Participants

The study subjects were 200 healthy subjects (HIV-negative) and 199 patients with HIV. Thus, the total sample consisted of 399 participants, with a mean age of 47.5 years (SD = 9.97). 

The total sample (*N* = 399) was made up of subjects diagnosed with HIV (*n* = 199) collected at the University Assistance Complex, specifically at Salamanca University Hospital. These patients voluntarily participated in the study after attending their scheduled appointment at the nursing clinic in the infectious disease unit. Therefore, this sub-sample was obtained with an incidental sampling, and is representative since the population of HIV subjects in the Salamanca University Hospital is approximately 600 patients.

After obtaining the sample of HIV subjects, the sample of healthy patients was selected (*n* = 200), following a sample by quotas method according to age, sex and level of equivalent studies, with the aim of achieving homogeneous sub-samples.

As inclusion criteria, it was highlighted that the subjects had to be of legal age and participate voluntarily in the project. In the case of subjects with HIV, it was also included to have a confirmed diagnosis of said disease. On the contrary, as an inclusion criterion in healthy patients, it was necessary to have not been diagnosed with HIV. Exclusion criterion included having any illness or medical or psychological disorder that prevented the subject from completing the study or signing the informed consent.

### 2.3. Data Collection

The data were collected from 4 February 2018 to 30 January 2020. The information was collected through nursing consultations, through self-administered questionnaires. Those subjects who agreed to participate in the study dedicated a time that ranged between 30 and 45 min to complete all the questionnaires, detailed below: 

#### 2.3.1. Sociodemographic Data Questionnaire

Sociodemographic data were collected through an instrument made up of a series of questions of a socio-demographic nature and information related to health. The variables collected through this questionnaire were:

- Health status (subjects without pathology, HIV-positive subjects)
- Socio-demographic variables studied (age, sex, marital status and educational level)

#### 2.3.2. Carol Ryff’s Psychological Well-Being Scale (PWBS)

Carol Ryff, in 1989, developed the Scales of Psychological Well-Being (SPWB). Diaz et al. (2006) adapted it to Spanish [30].

This author proposed a multidimensional model of psychological well-being consisting of six dimensions: self-acceptance, positive relations, autonomy, environmental mastery, purpose in life and personal growth. Van Dierendonck’s version consisted of a 39-item test with six answer options (from 1, totally disagree, up to 6, totally agree). The version proposed by Diaz and collaborators, used in this study, has 29 items. A total score is obtained for each dimension. Table 1 shows the psychometric properties of the Spanish version of this scale with 39 items (original) and with 29 items (version proposed by Díaz et al.). 

In this research, the use of the 29-item version was chosen for two reasons: (a) its brevity, and (b) its psychometric properties, which were better in the abbreviated version.

#### 2.3.3. Coping Strategies Questionnaire (Sandín and Chorot)

Sandín and Chorot in 2002 developed the scale of the Coping Strategies Questionnaire. This scale is based on a previous version: the Scale of Coping Strategies–Revised (EEC-R), created in 1999 by Sandín, Valiente and Chorot. The scale allows the study of seven dimensions of coping: social support seeking, overt emotional expression, religious coping, problem-solving coping, avoidance coping, negative auto-focused coping, and positive reappraisal. Two more general dimensions were performed after a second-order analysis. The first of them, emotional coping, included negative auto-focused coping and overt emotional expression. The other dimension, rational coping, included problem-solving coping, positive reappraisal and social support seeking. This questionnaire contains a scale comprising 42 items. Each item goes from 0 (never) to 4 (almost always) [31].

Table 2 shows the variance explained by each factor, as well as the associated Cronbach’s α coefficient. The total variance explained by the seven factors was 55.3%. Regarding the second order structure, with the two large groups of strategies (Rational and Emotional), 49.3% of the total variance of the test will be clarified.

#### 2.3.4. Resilience Scale (Wagnild and Young) 

Wagnild and Young in 1993 created The Resilience Scale. In 2002, Novella adapted the scale into Spanish [32]. This questionnaire contains a scale comprising 25 items, with a response range from 1 (totally disagree) to 7 (totally agree). 

The scale allows the study of five resilience factors: personal satisfaction, equanimity, feeling good alone, self-confidence, and perseverance. Regarding the psychometric properties, Table 3 shows the coefficients of each area obtained in the study by Novella (2002). The overall internal consistency measured through the Cronbach’s α coefficient had a value of 0.88. 

### 2.4. Ethical Considerations

The study was admitted by public institutions associated with the study. Addition-ally, it received a positive report from the Ethics Committee. The participation of the subjects was voluntary, ensuring the confidentiality and anonymity of the participants, who were given an information sheet and asked to complete the informed consent form.

### 2.5. Statistical Analysis 

Statistical analysis was performed using International Business Machines’ (IBM) Statistical Package for the Social Sciences (SPSS) v25 (IBM Corp., Armonk, NY, USA).

A descriptive analysis of the socio-demographic variables was carried out in terms of sample size and percentages. The analysis of the differences between the subsamples is presented. Due to the nature of the socio-demographic variables, Pearson’s χ^2^ test was used, using Cramer’s V to determine the effect size. The values proposed by Cohen (1988) were used to interpret the magnitude of the effect found.

To determine the differences in psychological well-being, resilience, and coping strategies between healthy subjects and HIV patients, the multivariate analysis of variance (MANOVA) was performed.

In all statistical tests, testing was significant when *p* < 0.05

## 3. Results

### 3.1. Descriptive Analysis

In total, 399 subjects participated in the study. Most of the study is made up of men (*N* = 293), and the subjects are mainly aged between 44 and 50 years (*N* = 116). The majority of the total sample is married/in a relationship (*N* = 182) and have an educational level equivalent to secondary or lower (*N* = 324) (Table 4). 

According to the health status of the patients, two categories are differentiated: HIV patients (*N* = 199) and healthy subjects (*N* = 200). No significant differences were obtained with respect to sex, educational level and age (*p* > 0.05). The only significant variable was marital status ($\chi^{2}$, = 49,881; *p* < 0.01). However, the effect is moderate (V = 0.354) (Table 4).

### 3.2. Resilience, Psychological Well-Being and Coping Strategies

Differences were observed between participant HIV-positive and healthy subjects. To determine the differences in resilience, psychological well-being and coping strategies between subjects with HIV and healthy participants, the MANOVA technique was applied. In relation to the multivariate test, in the Pillai trace it was observed that there were significant differences between groups (F(df) = 10.041 (18); *p* < 0.001). The power was high (1.000) and the size of the effect was large ($\eta_{p}{}^{2}$ = 0.323) (Table 5).

For the variables of resilience, significant differences were detected (Table 6): perseverance (F(df) = 14.699(1); *p* = <0.001) and self-confidence (F(gl) = 15.987(1); *p* = <0.001). In addition, significant differences were detected in all dimensions of psychological well-being, except in the dimension of autonomy (F(df) = 5.031(1); *p* = 0.025). The power was moderate (0.698), and the effect size was small ($\eta_{p}{}^{2}$ = 0.013). Finally, significant differences were found for the variables of coping: social support seeking (F(gl) = 32.381(1); *p* = <0.001), problem-solving coping (F(df) = 9.759(1); *p* = 0.002) and positive reappraisal. (F(df) = 51.520(1); *p* = <0.001).

Below, using the Dunnett, the differences according to the resilience factors between the groups are shown (Table 7). As can be seen, subjects with HIV, compared to healthy subjects, show lower scores in perseverance (HIV-Healthy = −1.90, STD. ERROR = 0.497, CI = [−3.08, −0.73]) and in self-confidence (HIV-Healthy = −2.25, STD. ERROR = 0.563, CI = [−3.58, −0.92]).

HIV subjects scored significantly less than healthy subjects in all well-being variables, except in autonomy (HIV-Healthy = −1.16, STD. ERROR = 0.518, CI = [−2.38, 0.06]). For the positive relationships variable, the one with the highest effect size, HIV subjects scored slightly more than four points less than healthy subjects (HIV-Healthy = −4.21, STD. ERROR = 0.475, CI = [−5.33, −3.08]) (Table 8).

In relation to coping strategies, it was found that HIV-positive participants use social support and problem-solving coping less frequently than healthy subjects (Table 9). In addition, patients with HIV used more negative auto-focused coping, compared to healthy patients (HIV-Healthy = 2.72, STD. ERROR = 0.379, CI = [−1.82, 3.61]). 

## 4. Discussion

The present study showed that participants with HIV had a significantly lower score than healthy subjects in the resilience factors: perseverance and self-confidence. 

This psychological variable is important because, as detailed in other investigations, there is enough published evidence that associates resilience with positive health benefits, favouring viral suppression in subjects with HIV [33] and positively impacting on their quality of life [34,35].

Other studies reflected that healthy subjects have higher levels of resilience than patients with chronic pathologies [13]. These results are in line with those obtained in our work. Similar results to those presented in our study are shown in the research by Mcgowan et al. (2018) [34], in which it was observed that the level of resilience was higher in healthy subjects than in those with a diagnosis of HIV. However, in contrast to our study, this difference was not significant. It should be noted that the presence of such research comparing the levels of resilience in HIV subjects and healthy subjects is very sparse. Therefore, we consider it necessary to develop more research in this area.

On the other hand, HIV-positive patients scored lower on all dimensions of psychological well-being compared to healthy participants, with the exception of the autonomy dimension. In a study by López et al. (2008) [36], it was suggested that patients with HIV have more psychological problems due to the stigma associated with the disease. This consideration could explain why HIV-positive participants in this study had lower levels of psychological well-being and resilience. There are not enough published projects that allow comparing the results obtained here in terms of the dimensions of psychological well-being. However, Wisk and Weitzman (2017) [37] in their study on subjects with chronic pathology and healthy subjects, observed that those individuals diagnosed with a chronic pathology had lower psychological well-being in adulthood than their healthy peers. In this way, we consider it necessary to detect which chronic pathologies are associated with lower psychological well-being. On the other hand, in a study by Chongwo et al. (2018) [38], it was evident that healthy subjects had better scores in terms of psychological well-being compared to the group of subjects with HIV. Additionally, in the study by Ramkisson et al. (2016), it was reflected that the absence of disease is associated with higher levels of psychological well-being than in subjects with HIV [39]. These findings are consistent with those of our study, where subjects diagnosed with HIV scored significantly less than healthy subjects in all dimensions of well-being, except for autonomy.

Finally, based on the coping strategies studied, it was observed that HIV subjects used strategies based on social support seeking and problem-solving coping less frequently than healthy subjects. Recalling the general classification of coping strategies, it is observed that patients with HIV use less rational strategies than participants without pathology. On the contrary, subjects with HIV used more emotional strategies, specifically in the dimension of negative self-centered coping, than healthy individuals.

These results are important because rational coping is associated with a greater ability of the patient to cope with the disease, achieving a greater adaptation to the disease and a greater degree of well-being. However, the styles of avoidance coping or emotional coping are related to negative psychological results, less adherence to treatment and worse adaptation to the disease, promoting levels of stress and anxiety [27]. Therefore, HIV-positive subjects could be using inappropriate strategies to cope with the disease. These results could be justified due to the great emotional impact of a HIV diagnosis [7]. Therefore, these patients do not feel able to actively face the consequences of the disease using strategies based on negative auto-focused coping. In addition, this greater use of emotional strategies could justify the lower levels of resilience and psychological well-being in this group of patients. 

However, other projects have highlighted that problem solving, seeking social support and avoidance were the coping strategies most used by HIV patients [40,41]. These statements are contrary to the results offered in this study, which reflects the need to continue research in this area. These results do not coincide with those found in our work, in which it was observed that the strategies based on social support seeking, problem-solving coping and avoidance coping were less used by HIV-positive participants compared to healthy subjects. In addition, the social support seeking strategy was the least used by patients with HIV compared to healthy subjects. As justification for this result, different investigations show that these patients experience more fear and feelings of shame, which can prevent these patients from turning to their relatives or friends, so they use this type of coping strategy less frequently [42,43,44]. Heydari el al. (2020), published a study similar to ours. The aim of this study was to compare the coping styles of people living with Human immunodeficiency virus, positive and negative, in the Iranian population. In this investigation it was detected that there were no differences in coping strategies between the subgroups [45]. These results are contrary to those presented in our study. However, it could be justified by the cultural difference between the samples of both studies.

It should be noted that there is little research focused on predicting the levels of resilience, psychological well-being and coping strategies used in subjects with HIV. In this way, our results show that it is necessary to develop more research that evaluates and compares resilience in these subjects, which allows us to determine the differences in resilience depending on the pathology.

Among the limitations of this study is the presence of significant differences in the sociodemographic variable of marital status between the two studied sub-samples. However, taking into account the high percentage of single subjects in the group of subjects with HIV, justified in part by the difficulties that this group may have in maintaining relationships, makes it very difficult to obtain homogeneous subsamples in this sociodemographic variable. In addition, with regard to the analysis of differences between sub-samples, marital status was the only significant variable and the size of the effect was small. Another limitation would be access to medical care, which would make it difficult to obtain a larger sample in a shorter period of time. Finally, we also highlight as a limitation the non-inclusion of the descriptive data of all the variables analyzed, as well as the values of resilience, psychological well-being and coping of each group. However, the purpose of the work was to compare the different health states with each other. Future investigations turned out to take into account the limitations indicated above to provide more specific evidence on the results found.

## 5. Conclusions

By way of conclusion, patients with HIV showed a lower score than healthy subjects in the factors of resilience, perseverance and self-confidence. In addition, they scored less than healthy subjects in all dimensions of psychological well-being except in autonomy. Finally, it was observed that subjects with HIV used rational coping strategies (problem-solving coping and social support seeking) less frequently than healthy subjects, although they used more negative auto-focused coping compared to the sub-sample without pathology.

It is highlighted that high levels of resilience, psychological well-being and rational coping strategies are related to a better prognosis and health benefits in HIV subjects. However, the present study found that patients with HIV have worse levels in these psychological constructs than healthy participants. 

Implications: These conclusions reflect the importance of the involvement of all health and mental health professionals who work with HIV patients, reflecting the need for a multidisciplinary team.

In addition, the development of interventions to promote resilience and active coping strategies can have a positive impact on the quality of life and well-being of people. Furthermore, it would be necessary to create public health measures that promote access to equality and reduce social stigma in HIV-positive patients.

## Figures and Tables

**Table 1:** Psychometric properties of the Psychological Well-being scale. Spanish version.

	39 Items Version		29 Items Version	
	Number of Items	α Cronbach	Number of Items	α Cronbach
Self-acceptance	6	0.83	4	0.84
Positive relations	6	0.81	6	0.78
Autonomy	8	0.73	6	0.70
Environmental mastery	6	0.71	5	0.82
Purpose in life	7	0.83	5	0.70
Personal growth	6	0.68	4	0.71

**Table 2:** Correlations between factors and Cronbach’s alpha coefficients.

	Variance Explained	α Cronbach
Social Support Seeking	20.3%	0.92
overt emotional expression	11.2%	0.74
Religious coping	7.8%	0.86
Problem-solving coping	5.3%	0.85
Avoidance coping	4.7%	0.76
Negative auto-focused coping	3.2%	0.64
Overt emotional expression	2.8%	0.71

**Table 3:** Reliability of the Resilience Scale.

	α Cronbach
Personal satisfaction	0.78
Equanimity	0.75
Feeling good alone	0.71
Self-confidence	0.80
Perseverance	0.76
Full scale	0.87

**Table 4:** Sociodemographic variables based on health status.

	HIV		Healthy		Total		Ji	TE	*p*
	N	%	N	%	N	%			
Nº participants	199	49.8%	200	50.2%	399	100%			
Sex									
Woman	48	24.1%	58	29.0%	106	26.5%	1.217	0.055	0.270
Man	151	75.9%	142	71.0%	294	73.5%			
Age									
43 years or younger	50	25.1%	59	29.5%	109	27.3%	8.509	0.146	0.037
44 to 50 years	61	30.7%	55	27.5%	116	29.1%			
From 51 to 55 years old	57	28.6%	38	19.0%	95	23.8%			
56 years or older	31	15.6%	48	24.0%	79	19.8%			
Civil status									
Married/couple	58	29.1%	124	62.0%	182	45.6%	49.881	0.354	0.000
Single/widowed/other	117	58.8%	51	25.5%	168	42.1%			
Separated/divorced	24	12.1%	25	12.5%	49	12.3%			
Level of studies									
Secondary or lower	168	84.4%	156	78.0%	324	81.2%	2.695	0.082	0.101
Superior	31	15.6%	44	22.0%	75	18.8%			

N: Number of subjects; %: percentage, $\chi^{2}:\;\mathrm{chi}-\mathrm{squared}$ TE: effect size; *p*: *p*-value.

**Table 5:** Multivariate tests: Pillai trace.

Effect	Value	F	Hypothesis df	Error df	*p*	$\eta_{p}{}^{2}$	Observed Power
Intersection	0.985	1411.971	18	379	<0.001	0.985	1.000
Health status	0.323	10.041	18	379	<0.001	0.323	1.000

$\eta_{p}{}^{2}:$ eta squared; df: degrees of freedom.

**Table 6:** Effects tests between subjects: health status.

Dependent Variable	F	Sig.	$\eta_{p}{}^{2}$	df	Observed Power
RS Personal satisfaction	4.323	0.038	0.011	1	0.545
RS Equanimity	1.971	0.301	0.003	1	0.178
RS Feeling good alone	13.389	0.110	0.006	1	0.358
RS Perseverance	14.699	<0.001	0.000	1	0.969
RS Self-confidence	15.986	<0.001	0.039	1	0.979
PWS Self-acceptance	43.336	<0.001	0.099	1	1.000
PWS Autonomy	5.031	0.025	0.013	1	0.698
PWS Purpose in life	73.559	<0.001	0.157	1	1.000
PWS Positive relations	78.408	<0.001	0.165	1	1.000
PWS Environmental mastery	29.122	<0.001	0.069	1	1.000
PWS Personal growth	36.523	<0.001	0.084	1	1.000
CS Problem-solving coping	9.759	0.002	0.024	1	0.876
CS Social support seeking	32.381	<0.001	0.076	1	1.000
CS Positive reappraisal	3.919	0.048	0.010	1	0.506
CS Negative auto-focused coping	51.520	<0.001	0.115	1	1.000
CS Overt emotional expression	2.571	0.110	0.006	1	0.359
CS Religious coping	1.199	0.274	0.003	1	0.194
CS Avoidance coping	257	0.263	0.003	1	0.201

$\eta_{p}{}^{2}$: eta squared; RS: resilience; PWS: psychological well-being; CS: coping strategies.

**Table 7:** Multiple comparisons: resilience factors.

Dependent Variable	(I) Subject Health Status	(J) Subject Health Status	Mean Difference (I-J)	Std. Error	CI 95%	
					Lower Bound	Upper Bound
RS Personal satisfaction	HIV	healthy subjects	−0.64	0.307	−1.36	0.09
RS Equanimity	HIV	healthy subjects	−0.26	0.248	−0.84	0.33
RS Feeling good alone	HIV	healthy subjects	−0.37	0.229	−0.91	0.17
RS Perseverance	HIV	healthy subjects	−1.90 *	0.497	−3.08	−0.73
RS Self-confidence	HIV	healthy subjects	−2.25 *	0.563	−3.58	−0.92

The Dunnett C test was used. * significant differences; RS: resilience; Std. error: standard error; CI: confidence interval.

**Table 8:** Multiple comparisons: dimensions of psychological well-being.

Dependent Variable	(I) Subject Health Status	(J) Subject Health Status	Mean Difference (I-J)	Std. Error	CI 95%	
					Lower Bound	Upper Bound
PWS Self-acceptance	HIV	Healthy subjects	−2.34 *	0.356	−3.18	−1.50
PWS Autonomy	HIV	healthy subjects	−1.16	0.518	−2.38	0.06
PWS Purpose in life	HIV	healthy subjects	−3.79 *	0.442	−4.83	−2.75
PWS Positive relations	HIV	healthy subjects	−4.21 *	0.475	−5.33	−3.08
PWS Environmental mastery	HIV	healthy subjects	−2.14 *	0.397	−3.08	−1.20
PWS Personal growth	HIV	healthy subjects	−1.94 *	0.321	−2.70	−1.18

The Dunnett C test was used. * significant differences; PWS: psychological well-being; Std. error: standard error; CI: confidence interval.

**Table 9:** Multiple comparisons: coping strategies.

Dependent Variable	(I) Subject Health Status	(J) Subject Health Status	Mean Difference (I-J)	Std. Error	CI 95%	
					Lower Bound	Upper Bound
CS Social support seeking	HIV	Healthy subjects	−3.73 *	0.655	−5.28	−2.18
CS Problem-solving coping	HIV	Healthy subjects	−1.61 *	0.516	−2.83	−0.39
CS Positive reappraisal	HIV	Healthy subjects	−0.89	0.452	−1.96	0.17
CS Negative auto-focused coping	HIV	Healthy subjects	2.72 *	0.379	1.82	3.61
CS Overt emotional expression	HIV	Healthy subjects	−0.62	0.385	−1.53	0.29
CS Religious coping	HIV	Healthy subjects	−0.71	0.647	−2.24	0.82
CS Avoidance coping	HIV	Healthy subjects	−0.52	0.462	−1.61	0.57

The Dunnett C test was used. * significant differences; CS: coping strategies; Std. error: standard error; CI: confidence interval.

## References

[1] Lamotte J.A. (2014). Infection due to HIV/aids in the current world. Medisan.

[2] Ministerio de Sanidad Consumo y Bienestar Social (2019). Vigilancia Epidemiológica del VIH y Sida en España 2018: Sistema de Información sobre Nuevos Diagnósticos de VIH y Registro Nacional de Casos de Sida.

[3] Cobos Manuel I., Jackson-Perry D., Courvoisier C., Bluntschli C., Carel S., Muggli E., Waelti Da Costa V., Kampouri E., Cavassini M., Darling K. (2020). Stigmatisation et VIH: Tous concernés [Stigma and HIV: Relevant for everyone]. Rev. Med. Suisse.

[4] Earnshaw V.A., Bogart L.M., Dovidio J.F., Williams D.R. (2015). Stigma and racial/ethnic HIV disparities: Moving toward resilience. Am. Psychol..

[5] Furlotte C., Schwartz K. (2017). Mental Health Experiences of Older Adults Living with HIV: Uncertainty, Stigma, and Approaches to Resilience. Can. J. Aging.

[6] Ruiz-Algueró M., Hernando V., Marcos H., Gutiérrez G., Pérez-Elías M.J., López-Bernaldo J.C., Pulido F., Górgolas M., Sanz J., Suarez-García I. (2021). Self-rated health among people living with HIV in Spain in 2019: A cross-sectional study. BMC Infect. Dis..

[7] Lessa T., Oliveira D.C., Marcos A., Tosoli A.M., Formozo G.A. (2014). Quality of life and people living with AIDS: Relationship with sociodemographic and health aspects. Rev. Lat. Am. Enferm..

[8] Nyongesa M.K., Nasambu C., Mapenzi R., Koot H.M., Cuijpers P., Newton C., Abubakar A. (2022). Psychosocial and mental health challenges faced by emerging adults living with HIV and support systems aiding their positive coping: A qualitative study from the Kenyan coast. BMC Public Health.

[9] Koerting A., Polo R., Vázquez M.C., Del Amo J. (2019). The Social Pact for the Non-discrimination and Equal Treatment Associated with HIV development. Rev. Española Salud Pública.

[10] Earnshaw V.A., Lang S.M., Lippitt M., Jin H., Chaudoir S.R. (2015). HIV Stigma and Physical Health Symptoms: Do Social Support, Adaptive Coping, and/or Identity Centrality Act as Resilience Resources?. AIDS Behav..

[11] Yang X., Li X., Qiao S., Li L., Parker C., Shen Z., Zhou Y. (2020). Intersectional stigma and psychosocial well-being among MSM living with HIV in Guangxi, China. AIDS Care.

[12] Plascencia de la Torre J.C., Castellanos C. (2019). Resilience assessment in mexicans diagnosed with hiv: A comparative study. Salud Soc..

[13] Gheshlagh R., Sayehmiri K., Ebadi A., Dalvandi A., Dalvand S., Nourozi K. (2016). Resilience of Patients With Chronic Physical Diseases: A Systematic Review and Meta-Analysis. Iran. Red Crescent Med. J..

[14] Gheshlagh R.G., Ebadi A., Dalvandi A., Rezaei M., Tabrizi K.N. (2017). A Systematic Study of Resilience in Patients with Chronic Physical Diseases. Nurs. Midwifery Stud..

[15] Quiceno M., Vinaccia S. (2012). Resilience and sociodemographic characteristics in the chronic ill. Psicol. Desde. Caribe..

[16] Fernanda S., Ribeiro L., Eugenia M., Borreto M. (2015). Resilience in chronic diseases: A systematic review. Cogent Psychol..

[17] Min J., Yoon S., Lee C., Chae J., Lee C., Song K., Kim T. (2013). Psychological resilience contributes to low emotional distress in cancer patients. Support Care Cancer.

[18] Crowley T., Van der Merwe A.S., Esterhuizen T., Skinner D. (2021). Resilience of adolescents living with HIV in the Cape Metropole of the Western Cape. AIDS Care.

[19] Amar J., Martínez M., Utria L. (2013). New approach to health considering the resilience. Salud Uninorte..

[20] Gonzalez I., Eduarda D.S., Paiva L., Rossi L.A., Dantas S., Alcalá D. (2016). Anxiety, depression, resilience and self-esteem in individuals with cardiovascular diseases. Rev. Lat.-Am. Enfermagem..

[21] Quiceno J.M., Vinaccia S. (2011). Resilience: A perspective from chronic disease in the adult population. Pensam. Psicol..

[22] Schumacher A., Sauerland C., Silling G., Berdel W.E., Stelljes M. (2014). Resilience in patients after allogeneic stem cell transplantation. Support Care Cancer.

[23] Yang Z., Wang J., Zhang B., Zeng Y., Ma H. (2014). Factors influencing resilience in patients with burns during rehabilitation period. Int. J. Nurs. Sci..

[24] Babić R., Babić M., Rastović P., Ćurlin M., Šimić J., Mandić K., Pavlović K. (2020). Resilience in Health and Illness. Psychiatr. Danub..

[25] Vázquez C., Hervás G., Rahona J.J., Gómez D. (2009). Psychological well-being and health: Contributions from Positive Psychology. Anu. Psicol. Clínica Salud.

[26] Orozco-Gómez Á., Castiblanco-Orozco L. (2015). Psychosocial Factors and Psychological Intervention in Chronic Non-Communicable Disease. Rev. Colomb. Psicol..

[27] Carrobles J.A., Remor E., Rodríguez-Alzamora L. (2003). Coping, perceived social support and emotional distress in patients with HIV infection. Psicothema.

[28] Milena G.A., Quiceno J.M., Vinaccia S., Martínez L.A., Otalvaro M.C. (2009). Copying Strategies, Anxiety and Depression in HIV/Aids Patients. Ter. Psicológica.

[29] Garrido-Hernansaiz H., Murphy P.J., Alonso-Tapia J. (2017). Predictors of Resilience and Posttraumatic Growth Among People Living with HIV: A Longitudinal Study. AIDS Behav..

[30] Díaz D., Rodríguez-Carvajal R., Blanco A., Moreno-Jiménez B., Gallardo I., Valle C., Van Dierendock D. (2006). Adaptation of the Ryff psychological well-being scales. Psicothema.

[31] Sandín B., Chorot B. (2002). The Coping Strategies Questionnaire: Development and preliminary validation. Rev. Piscopatoogía Piscologia Clínico.

[32] Novella A. (2002). Incremento de la Resiliencia Luego de la Aplicación de un Programa de Psicoterapia Breve en Madres Adolescentes. Ph.D. Thesis.

[33] Dale S.K., Cohen M.H., Kelso G.A., Cruise R.C., Weber K.M., Watson C., Burke-Miller J.K., Brody L.R. (2014). Resilience Among Women with HIV: Impact of Silencing the Self and Socioeconomic Factors. Sex Roles.

[34] Mcgowan J.A., Brown J., Lampe F.C., Lipman M., Smith C., Rodger A. (2018). Resilience and Physical and Mental Well-Being in Adults with and Without HIV. AIDS Behav..

[35] Brown L.L., Martin E.G., Knudsen H.K., Gotham H.J., Garner B.R. (2021). Resilience-Focused HIV Care to Promote Psychological Well-Being during COVID-19 and Other Catastrophes. Front. Public Health.

[36] López M., Laviana M., Fernández L., López A., Rodríguez A.M., Aparicio A. (2008). The struggle against the stigma and discrimination in mental health. A complex strategy based on available data. Rev. Assoc. Española Neuropsiquiatría.

[37] Wisk L.E., Weitzman E.R. (2017). Expectancy and Achievement Gaps in Educational Attainment and Subsequent Adverse Health Effects Among Adolescents with and without Chronic Medical Conditions. J. Adolesc. Health.

[38] Chongwo E., Ssewanyana D., Nasambu C., Mwangala P.N., Mwangi P.M., Nyongesa M.K., Newton C.R., Abubakar A. (2018). Validation of a Swahili version of the World Health Organization 5-item well-being index among adults living with HIV and epilepsy in rural coastal Kenya. Glob. Health Res. Policy.

[39] Ramkisson S., Pillay B.J., Sartorius B. (2016). Anxiety, depression and psychological well-being in a cohort of South African adults with Type 2 diabetes mellitus. South Afr. J. Psychiatry.

[40] Grant N., Tarakeshwar N., Hansen N., Kochman A., Sikkema K. (2009). Coping Mediates Outcome Following a Randomized Group Intervention for HIV-Positive Bereaved Individuals. J. Clin. Psychol..

[41] Xiao Z., Li X., Qiao S., Zhou Y., Shen Z. (2018). Coping, social support, stigma, and gender difference among people living with HIV in Guangxi, China. Psychol. Health Med..

[42] Choi K.H., Gibson D.R., Han L., Guo Y. (2004). High levels of unprotected sex with men and women among men who have sex with men: A potential bridge of HIV transmission in Beijing, China. AIDS Educ. Prev..

[43] Wong W.C.W., Zhang J., Wu S.C., Kong T.S.K., Ling D.C.Y. (2006). The HIV related risks among men having sex with men in rural Yunnan, China: A qualitative study. Sex. Transm. Infect..

[44] Ye Z., Chen L., Lin D., Wang S.X. (2018). The Relationship between Posttraumatic Stress Disorder Symptoms and Posttraumatic Growth among HIV-Infected Men who Have Sex with Men in Beijing, China: The Mediating Roles of Coping Strategies. Front. Psychol..

[45] Heydari M., Karimzadeh Y., Faghih M., Heydari Z., Hosseini E., Mehraeen M. (2020). Coping styles in HIV positives and HIV negatives. BMC Psychol..

